# Surface proteins modulate extracellular vesicle mediated inhibition of Aβ(1‐42) aggregation

**DOI:** 10.1002/pro.70849

**Published:** 2026-11-05

**Authors:** Vesa Halipi, Georgia Daoutsali, Fritjof Havemeister, Viktoria de Carvalho, André Görgens, Samir El Andaloussi, Elin K. Esbjörner

**Affiliations:** ^1^ Division of Molecular Bioscience, Department of Life Sciences Chalmers University of Technology Gothenburg Sweden; ^2^ Division of Biomolecular and Cellular Medicine, Department of Laboratory Medicine Karolinska Institutet Stockholm Sweden; ^3^ Department of Cellular Therapy and Allogeneic Stem Cell Transplantation (CAST) Karolinska University Hospital Huddinge Sweden; ^4^ Karolinska ATMP Center, Karolinska Institutet, ANA Futura Huddinge Sweden

**Keywords:** Alzheimer's disease, amyloid kinetics, amyloid‐β, EVs, extracellular vesicles, protein aggregation

## Abstract

Aggregation of the amyloid‐β (Aβ) peptide into amyloid oligomers and fibrils is a central pathogenic feature and therapeutic target in Alzheimer's disease (AD). Understanding the aggregation reaction and how it is modulated by the biological environment is therefore important. Cell‐secreted extracellular vesicles (EVs) are emerging as modulators of Aβ aggregation, transport and toxicity. Here, we show that EVs from a variety of human and non‐human cells have highly similar retarding effects on the rate of Aβ(1‐42) fibril formation, suggesting that aggregation inhibition is a common EV feature. Furthermore, we examine how different EV biomolecular classes contribute to shape this effect. We show that enzymatic removal of surface‐associated EV proteins significantly enhances their ability to inhibit aggregation. Similar, albeit considerably smaller, alterations were observed upon removal of cell surface protein‐associated glycans. This suggests that EV‐mediated inhibition of Aβ(1‐42) aggregation may primarily be regulated by lipids and that surface proteins function to modulate this effect, through steric hindrance and/or direct interactions with Aβ(1‐42) species. We further observed a minor but statistically robust enhancement of EV‐mediated Aβ(1‐42) aggregation inhibition upon removal of proteoglycans, suggesting that carbohydrates on the EV‐surface also contribute to modulate their inhibitory effect. Altogether, these observations contribute mechanistic insight into the role of EVs as modulators of Aβ aggregation and highlight their possible contributions to maintenance of extracellular protein homeostasis.

## 1 INTRODUCTION

Alzheimer's disease (AD) is a neurodegenerative disorder characterized by the aggregation of amyloid‐beta (Aβ) peptides into insoluble fibrils that accumulate in the brain as extracellular plaque deposits (Tiwari et al., 2019). Preceding plaque formation, smaller soluble Aβ aggregates such as oligomers and short fibrils accumulate in both intracellular and extracellular locations of the brain (Takahashi et al., 2017) and their presence has been associated with neurotoxicity and neuronal degeneration in AD (Haass & Selkoe, 2007; Hampel et al., 2021; Lue et al., 1999; Scheltens et al., 2021). Antibody treatments targeting such Aβ aggregates have recently gained clinical approval due to their ability to moderately slow down early‐stage disease progression (van Dyck et al., 2023). However, many Aβ‐targeting drugs in clinical trials face challenges with target specificity and toxic side effects (Zhang et al., 2023), and their efficacy needs to be improved, emphasizing the importance of a better understanding of the mechanisms of Aβ aggregation and accumulation, as well as how these reactions are modulated by biological factors in the diseased brain.

Aβ is a proteolytic cleavage product of the membrane‐bound amyloid precursor protein (APP) and exists as several isoforms of which the 42‐residue variant Aβ(1‐42) is known to be especially aggregation‐prone and neurotoxic (Jarrett et al., 1993) whereas the shorter Aβ(1‐40) variant is more abundant (Gravina et al., 1995). Biophysical studies of the self‐assembly of various monomeric Aβ isoforms into amyloid fibrils have provided important insights into the molecular mechanisms of their fibrillation (Jan et al., 2011; Meisl et al., 2014; Scheltens et al., 2021; Selkoe & Hardy, 2016). Aβ(1‐42) has been defined to follow a secondary nucleation‐dominated process (Cohen et al., 2013) in which the auto‐catalytic formation of new amyloid seeds on the surface of existing fibrils strongly dictates not only the rate of aggregate formation but also the generation of toxic oligomers (Cohen et al., 2013; Meisl, Yang, et al. 2016). Kinetic analysis of the modes of action of therapeutic anti‐amyloid antibodies has furthermore indicated the significance of inhibiting the secondary nucleation step to achieve Aβ(1‐42) aggregate suppression (Linse et al., 2020). On the other hand, it has been shown that fibrillation of Aβ(1‐42) peptides carrying the aggregation‐enhancing arctic mutation (E22G), which is associated with early onset AD, is instead dominated by fibril fragmentation (Bolognesi et al., 2014), suggesting that there is significant context‐dependent modulation of the aggregation pathway and that this can affect both rates and morphological characteristics of ensuing aggregates, including their size.

Aβ aggregation is strongly modulated by the physicochemical conditions of the microenvironment (Cohen et al., 2015; Meisl, Yang, et al., 2016). Lipid membranes are critical in this context, both because of their omnipresence in subcellular and extracellular locations where Aβ peptides are abundant, including the endolysosomal system (Gouras et al., 2000) and synaptic cleft (Sokolow et al., 2012), and because of the links between dysregulation of lipid homeostasis in the brain and AD pathology (Di Paolo & Kim, 2011). Biophysical studies have shown that various brain‐abundant membrane lipids, including cholesterol, sphingomyelin, ganglioside GM1, and phospholipids, can modulate various mechanistic steps in Aβ aggregation (Amaro et al., 2016; Habchi et al., 2018; Lindberg et al., 2017; Owen et al., 2018), resulting in both enhanced (Habchi et al., 2018; Lindberg et al., 2017; Owen et al., 2018) and inhibited (Amaro et al., 2016) fibrillation. The outcome of membrane‐mediated regulation of Aβ aggregation is therefore likely dependent on the complexity and composition of the biological membrane (Sanguanini et al., 2020; Sasanian et al., 2025) and may, in a biological context, be further modulated by membrane‐associated proteins and carbohydrates.

We recently demonstrated that cell‐secreted, so‐called extracellular vesicles (EVs) slow down Aβ(1‐42) aggregation by interfering with the amyloid fibril elongation step and that their presence thereby diverts the aggregation outcome towards short fibrils (Halipi et al., 2024). EVs are high complexity cell‐derived lipid vesicles that are increasingly recognized as important mediators of intercellular and inter‐organ communication (Abels & Breakefield, 2016; van Niel et al., 2018), also in the brain (Basso & Bonetto, 2016). They have been implicated as carriers of misfolded and aggregated proteins in several neurodegenerative disorders (Hill, 2019), including as transporters of Aβ (Vandendriessche et al., 2020) as well as APP (van Niel et al., 2018; Welsh et al., 2024) in AD (Vandendriessche et al., 2020) and are suggested to contribute to prion‐like propagation of protein aggregation pathologies (Vingtdeux et al., 2012).

Interestingly, we found that EVs from both neuroblastoma SH‐SY5Y and non‐neuronal human embryonic HEK293‐T cells exhibited highly similar inhibitory effects on Aβ(1‐42) aggregation despite the marked difference in cell source (Halipi et al., 2024), suggesting that the aggregation modulatory effect emanates from some common compositional feature of the EVs rather than brain‐specific components. Moreover, these EV types were found to slow down fibril elongation (i.e., the addition of monomers to fibril ends), rather than affecting primary or secondary nucleation as seen in most biophysical studies with synthetic lipid membranes (Lindberg et al., 2017; Owen et al., 2018). This suggests that not only the lipid portion, but also the surface proteins and carbohydrates that are associated with EVs play an important role.

This study further dissects the origin of EV‐mediated inhibitory effects on the aggregation of Aβ(1‐42). We have examined EVs from a wider variety of cell sources but also used neuroblastoma‐derived (SH‐SY5Y) EVs to explore in more detail the role of EV surface proteins and protein‐attached sugars (proteoglycans). These two biomolecular classes are, alongside lipids, important for EV functionalization (Jeppesen et al., 2014) affecting properties such as EV cell uptake and biodistribution (Cerezo‐Magana et al., 2020; Christianson et al., 2013; Yanez‐Mo et al., 2015). Proteoglycans, particularly those with heparan sulfate moieties, have, furthermore, been extensively studied in the context of AD (van Horssen et al., 2003) and been shown to co‐deposit with Aβ in plaques (Snow et al., 1994). We (Wesen et al., 2018) and others (Sandwall et al., 2010; Snow et al., 1990) have reported that proteoglycans, furthermore, promote Aβ cellular accumulation and regulate Aβ cytotoxicity.

This study extends our observation that EVs, regardless of cell source, slow down the in vitro aggregation of monomeric Aβ(1‐42). Furthermore, we show that enzymatic digestion of EV surface proteins leads to a significant enhancement of this inhibitory effect, suggesting that these proteins have a marked and important regulatory role. Removal of surface‐associated carbohydrates also contributes to the aggregation‐modulatory effect of EVs, albeit to a lesser extent than proteins. Altogether, this study provides new insights into how EVs, through their surface composition, shape interaction with Aβ peptides and thus can tune aggregation outcomes. These findings extend our current understanding of how EVs may contribute to modulating Aβ pathology and hence their role in AD.

## 2 RESULTS

### 2.1 EVs from diverse cell sources slow down Aβ(1‐42) aggregation

In previous work, we showed that EVs from both neuronal and non‐neuronal cells inhibited Aβ(1‐42) aggregation by an elongation inhibitory mechanism (Halipi et al., 2024). To explore the generality of this observation, we here examined additional EV sources, prepared from cultures of, respectively, umbilical cord‐blood derived mesenchymal stromal (MSC) cells, human monocytic THP‐1 cells, and Jurkat T‐cells. MSC‐derived EVs have been shown to exert immunomodulatory and neuroprotective effects in AD mouse models (Losurdo et al., 2020; Wang & Yang, 2021), whereas the THP‐1 and Jurkat EVs were chosen because of their proposed links to neuroinflammation (Minter et al., 2016), microglia activation, and proinflammatory cytokine release (McGeer et al., 1987; Udan et al., 2008), conditions which are associated with AD. Prior to aggregation experiments, the EV samples were analyzed using Nanoparticle Tracking Analysis (NTA) to determine particle size and concentration (Figures 1 and S1), confirming a size distribution within the expected size range for small EVs (~50–200 nm [Jia et al., 2022]).

**FIGURE 1:**
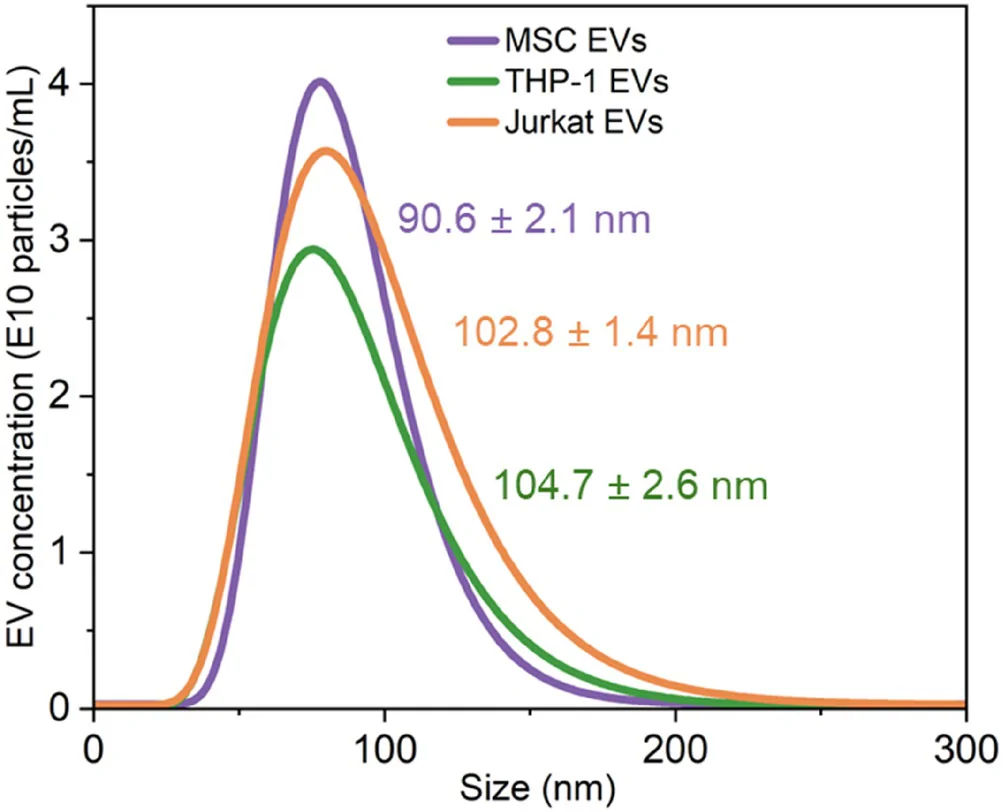
EV size distributions. Size distributions based on nanoparticle tracking (NTA) analysis for EVs derived from cord‐blood derived MSC, THP‐1, and Jurkat cells. The mean particle size in the different EV samples is shown as diameter ± standard deviation. The figure shows the distributions as nonlinear lognormal curves fitted to the raw NTA data, which can be found in Figure S1.

The effects of the three EV types on the kinetics of Aβ(1‐42) fibril formation were analyzed using Thioflavin‐T (ThT) fluorescence monitored kinetic assays (Figure 2a–c). This revealed that all EVs slowed down Aβ(1‐42) aggregation in a concentration‐dependent manner, consistent with our previous work (Halipi et al., 2024). An approximate three‐fold increase in aggregation half‐times (*t*_half_) was observed at the highest EV concentration (7.2e9 particles/mL) tested (Figure 2d).

**FIGURE 2:**
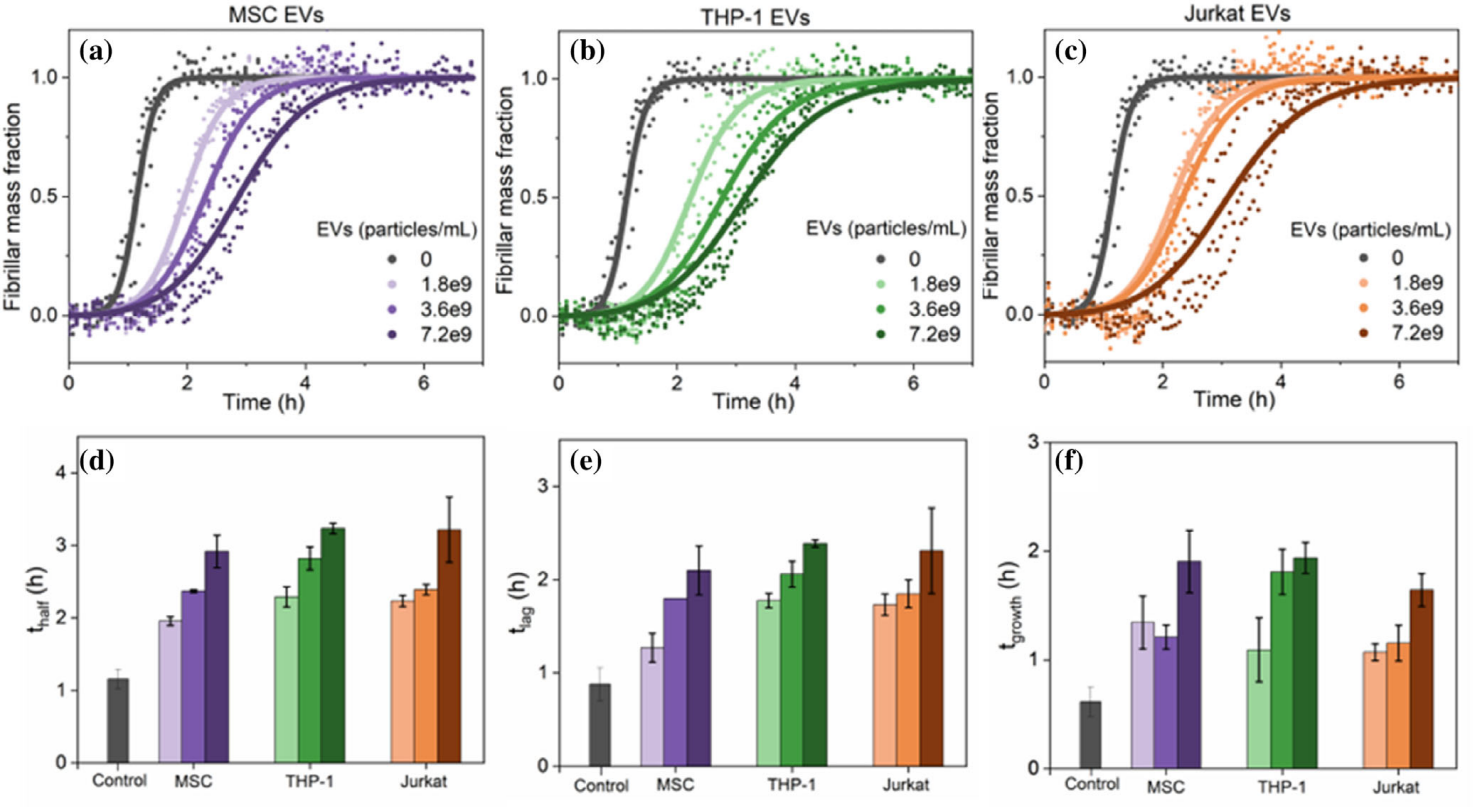
Aβ(1‐42) aggregation kinetics in the presence of cord‐blood derived MSC, THP‐1, and Jurkat EVs. (a–c) Normalized ThT fluorescence assay over time showing Aβ(1‐42) aggregation (2 μM) in absence or presence of indicated EV concentrations isolated from (a) umbilical cord‐blood derived MSCs, (b) THP‐1 cells, and (c) Jurkat cells. The dotted lines are experimental data points, and the solid lines show fittings of the experimental data using a multi‐step secondary nucleation dominated model for amyloid formation setting the combined rate constants for elongation and secondary nucleation (*k*_+_ *k*_2_) as a free parameter, as described in the main text and in Section 4. (d–f) Reaction half‐times (*t*_half_) (d), lag‐times (*t*_lag_) (e), and growth‐times (*t*_growth_) (f). The error bars represent the standard deviation (*n* = 3). Statistics: Two‐way ANOVA for factors EV type and concentration; see full ANOVA results in Tables S1–S3.

The aggregation kinetic curves were further analyzed by extracting lag‐times (*t*_lag_, defined as the reaction time for 10% of the total fibrillar content to form) and growth‐times (*t*_growth_, defined as the reaction time to increase the fibril content from 10% to 90% of the total fibrillar content) (Figure 2e,f). Although the magnitude and concentration‐dependence of all these parameters were largely similar across the three EV types, two‐way analysis of variance (ANOVA, Tables S1–S3) suggested statistically significant differences between EV types for all kinetic parameters (*t*_half_, *t*_lag_, and *t*_growth_; *p* < 0.01, Tables S1–S3). This indicates subtle differences in their effects, likely relating to their compositions. Moreover, comparison of the *t*_half_ data in Figure 2d to our previously obtained data for human neuroblastoma derived SH‐SY5Y and human embryonic kidney HEK293‐T EVs (Halipi et al., 2024) shows that even though the inhibitory trend is highly consistent, this new panel of EVs is somewhat less effective, even after data has been compensated for slight variations in overall aggregation speed (Figure S2). Altogether, these observations support the hypothesis that EVs have a generic ability to slow down Aβ(1‐42) aggregation but also highlight some variability suggesting that their exact biomolecular composition is also important.

In previous work, we established that EVs from SH‐SY5Y and HEK293‐T cells inhibit Aβ(1‐42) fibril elongation, based on kinetic modeling of seeded and unseeded aggregation data and the observation that EVs reduced fibril length (Halipi et al., 2024). Here, we further analyzed the aggregation kinetics by fitting a multi‐step secondary nucleation model to the kinetic data, using the online Amylofit tool (Meisl, Kirkegaard, et al., 2016) as described in Section 4. The fitted curves are shown as solid lines in Figures 2a–c and S3a–c. We used a procedure with sequential fitting where one of the two combined rate constants in the model (*k*_+_ *k*_*n*_ which is the product of elongation and primary nucleation rates, or *k*_+_ *k*_2_ which is the product of elongation and secondary nucleation rates (*k*_+_ *k*_2_)) were kept constant to explore how well the inhibitory effect of EVs could be explained by variation in the other (free) rate constant (Sasanian et al., 2025). This identified inhibition of secondary aggregation pathways (*k*_+_ *k*_2_) as the dominant aggregation‐retarding effect, evident both from visual inspection of the fit (Figure 2a–c vs. Figure S3a–c) and comparison of mean residual errors (MRE) (Tables S4–S6). Although it is not possible to decouple effects on secondary nucleation and elongation using this data analysis approach, the change in shape of the kinetic curves with extensions of both *t*_lag_ and *t*_growth_ (Figure 2e,f) is highly similar to our previous work (Halipi et al., 2024) and in line with the expected behavior of elongation inhibitors (Munke et al., 2017). Furthermore, the conclusion is supported by observations that EVs from the MSC, THP‐1 and Jurkat cells shortened Aβ(1‐42) fibrils (see below).

Having established that EVs of different cellular origins share a common mechanistic ability to slow down Aβ(1‐42) fibril formation, we proceeded to analyze Aβ(1‐42) fibril morphology using Atomic Force Microscopy (AFM). Figures 3a–d and S4 show representative images of mica‐deposited, dried, Aβ(1‐42) fibrils taken at the endpoints of kinetic experiments. The images confirm the formation of fibrils both in the absence of EVs (buffer) and in the presence of EVs at a concentration of 7.2e9 particles/mL (the highest concentration tested), consistent with the observed enhancement of ThT fluorescence (Figure 2). In addition, small spherical particles appeared in EV‐containing samples (Figure 3b–d) suggesting possible formation of oligomers. This was not observed in our previous work (Halipi et al., 2024). However, we also noted that the background in EV‐containing samples was coarser than for Aβ(1‐42) alone, suggesting that EVs may have adsorbed and ruptured on the mica surface during drying (white square in Figure 3d and Figure S5).

**FIGURE 3:**
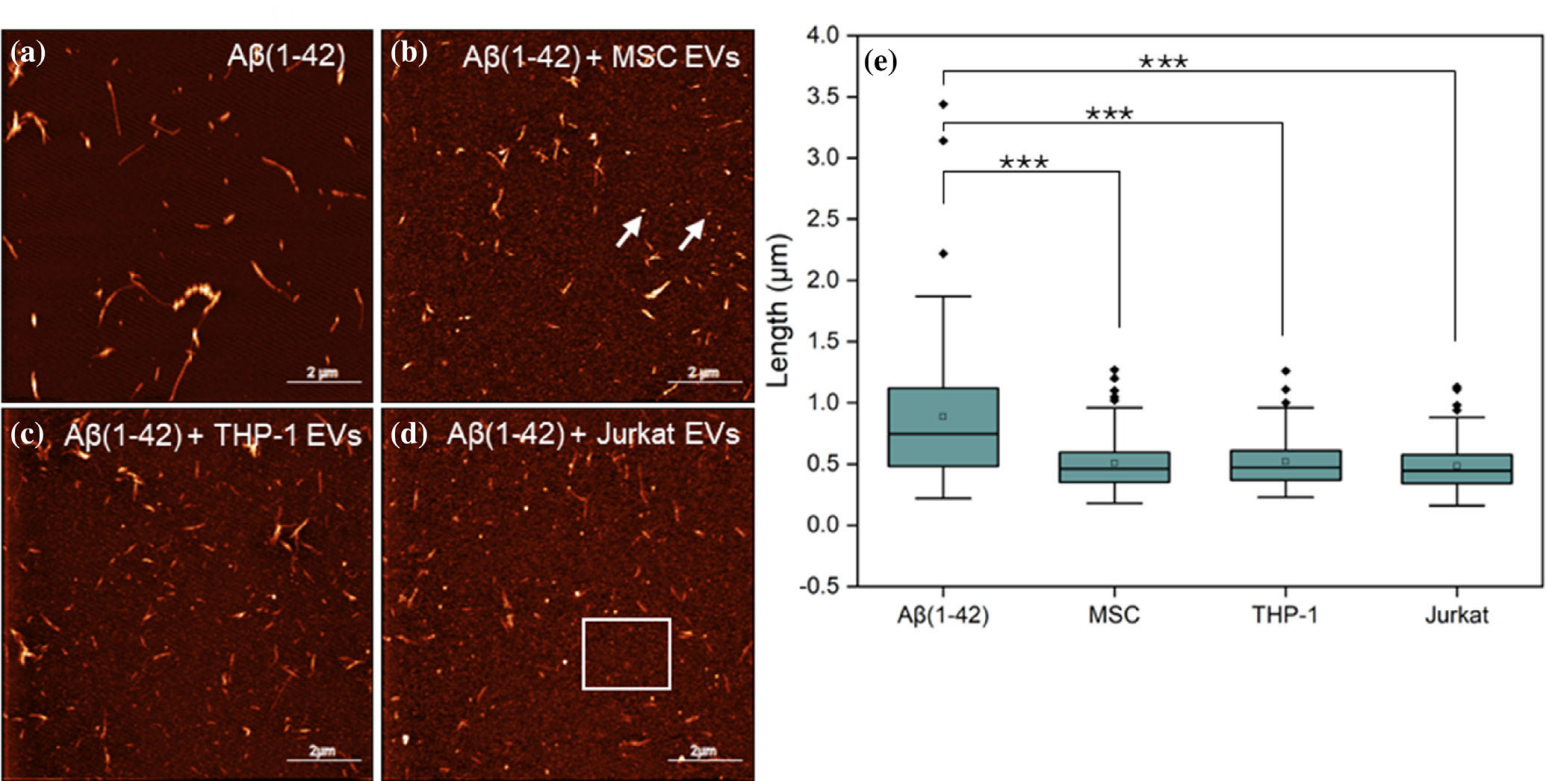
Morphological analysis of Aβ(1‐42) fibrils formed in absence and presence of EVs. (a–d) Representative AFM images of Aβ(1‐42) aggregation (2 μM) in absence (a) or presence of EVs from (b) umbilical cord‐blood derived MSCs, (**c**) THP‐1 cells, and (d) Jurkat cells. Scale bars = 2 μm. White arrows indicate smaller aggregate species in EV samples, and the white square marks the altered surface background in presence of EVs. (e) Analysis of fibril length based on AFM images (see Section 4) of Aβ(1‐42) fibrils (mean ± standard deviation, *n* = 100 fibrils from 11 images for the Aβ(1‐42) + THP‐1 EVs condition and from 10 images for the other conditions, *** denotes *p* < 0.001, by one‐way ANOVA and Post hoc Bonferroni‐corrected means comparison tests) formed in absence and presence of the indicated EVs. The EV concentration was 7.2e9 particles/mL in all conditions containing EVs. Box plots represent the interquartile range (25–75th percentile), mean values are presented as squares, median values as horizontal lines. Whiskers indicate values within 1.5× the interquartile range and black diamonds show outliers.

The AFM images were used to estimate fibril lengths (Figure 3e) and cross‐sectional heights (Figure S6). While the Aβ(1‐42) fibrils formed in the presence of EVs showed small variations in cross‐sectional heights compared to fibrils formed in buffer, fibril lengths were significantly altered. The mean lengths of fibrils were 506 ± 23 nm for fibrils formed in the presence of MSC‐derived EVs, 518 ± 20 nm in the presence of THP‐1‐derived EVs, and 481 ± 20 nm in the presence of Jurkat‐derived EVs, compared to 886 ± 57 nm for Aβ(1‐42) fibrils formed in buffer. This corresponds to a fibril length reduction of ca 45% and is a clearly significant effect (one‐way ANOVA, *p* < 0.001), consistent with our previous reports where EVs were shown to reduce fibril length by 40% (Halipi et al., 2024). Small differences in mean fibril lengths were observed among the different samples, but these were not statistically significant (post hoc Bonferroni‐corrected means comparison *p* > 0.99). Notably, the observation of EVs reducing fibril length supports the above conclusion of interference with the elongation step, as inhibition of secondary nucleation (which would be the other possible effect agreeing with the fitting of kinetic data) would instead significantly limit the number of aggregates formed and hence drive generation of longer fibrils under conditions where the total peptide concentration is constant. Altogether, by extending kinetic and morphological analysis of Aβ(1‐42) to a broader panel of EVs, this study supports the idea that EVs, independently of their cellular origin, slow down Aβ(1‐42) aggregation by a common mechanism.

### 2.2 Surface proteins modulate the aggregation‐inhibitory effect of EVs

EVs have a diverse surface proteome consisting of both integral and peripheral membrane proteins, and other adsorbed proteins (Hallal et al., 2022). The membrane‐associated protein content can amount to up to 50% (w/w) or more of the EV membrane (Osteikoetxea et al., 2015) and may, to a large extent, cover the EV surface (Hallal et al., 2022). We therefore hypothesized that they must contribute to modulate Aβ(1‐42) aggregation. To test this idea, we used EVs from SH‐SY5Y neuroblastoma cells, which are common neurodegenerative disease cell models (Agholme et al., 2010; Xicoy et al., 2017) and established a protocol to enzymatically digest their surface proteins using trypsin. The trypsin‐treated EVs were separated from trypsin and released protein fragments using ultrafiltration. The removal was first verified by NTA, showing that trypsin‐treated EVs had ~8 nm smaller diameter than control EVs (Figure 4a, Table S7). This corresponds to a ~4 nm thinning of the EV leaflet and is comparable to size reductions following enzymatic protein digestion reported by others (Choi et al., 2020; Shtam et al., 2018; Silva et al., 2025; Skliar et al., 2018). Importantly, the trypsin treatment did not significantly reduce EV concentration (Figure S7), suggesting that their membranes remained intact.

**FIGURE 4:**
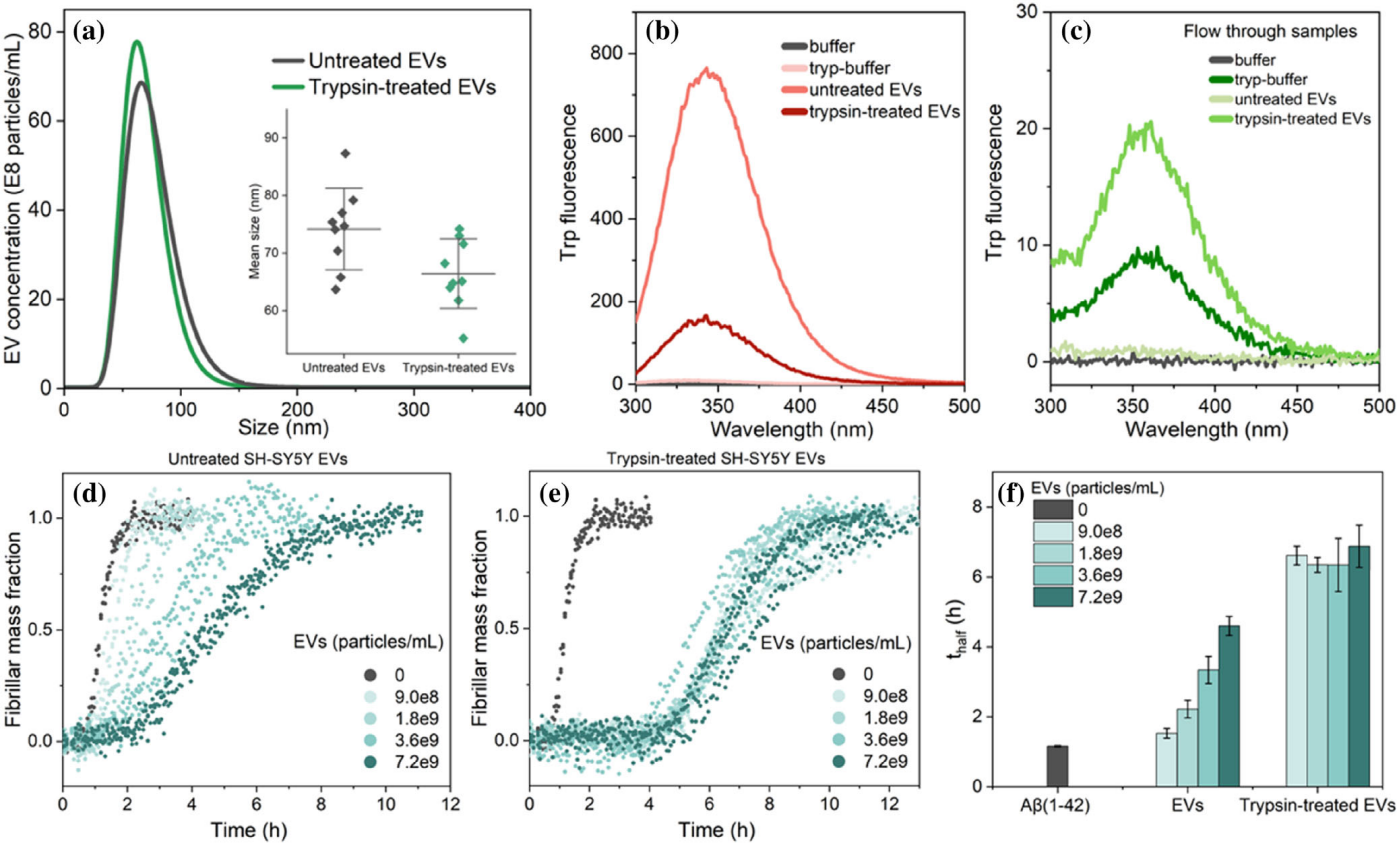
Characterization of EV surface protein digestion after trypsin‐treatment and their effect on Aβ(1‐42) aggregation kinetics. (a) Size distributions and concentrations of SH‐SY5Y EVs, untreated EVs (black) and trypsin‐treated EVs (green) as determined by NTA. Curves show one representative NTA measurements while distribution plots show mean size of EVs ± SD per group (*n* = 9). (b and c) Tryptophan (and tyrosine) fluorescence of EV samples (b) and flow through (c) after trypsin‐treatment and removal of peptide fragments and trypsin with ultrafiltration (see Section 4). Note that the absolute intensities in (b) and (c) cannot be compared because of the substantial (>20‐fold) dilution during ultrafiltration. (d and e) Normalized aggregation kinetic curves of 2 μM Aβ(1‐42) in absence or presence of untreated (d) vs. trypsin‐treated (e) SH‐SY5Y EVs on Aβ(1‐42) aggregation kinetics. (f) Reaction half‐times (*t*_half_) as a function of EV concentration, derived from the data in (d and ed).

To further confirm that the trypsin treatment and subsequent trypsin removal was effective, we analyzed the intrinsic protein fluorescence emanating from tryptophan and tyrosine in the ultrafiltrated EV samples (Figure 4b) as well as the flow‐through (Figure 4c) using fluorescence spectroscopy. Control experiments where trypsin solutions with the same concentration used for digestion were ultrafiltrated in absence of EVs verified that trypsin was effectively removed (Figure S8). Next, we examined EV samples and found that the level of intrinsic fluorescence was ~80% lower in the trypsin‐treated EV sample compared to the untreated EV control (Figure 4b). Moreover, the fluorescence spectra had emission maxima around 340 nm, which is consistent with the spectral signature of tryptophan in folded or membrane‐associated proteins (Lakowicz, 1988). Furthermore, the flow‐through of the ultrafiltrated trypsin‐treated EV sample showed substantial fluorescence in combination with a significant spectral red‐shift, consistent with the presence of unfolded fragments with solvent‐exposed tryptophan residues in this fraction, whereas the corresponding flow‐through for non‐trypsinized EVs showed negligible fluorescence (Figure 4c).

Using ThT kinetic assays, we found that trypsin‐treated EVs had a markedly stronger inhibitory effect on Aβ(1‐42) aggregation than untreated EVs. They also significantly altered the shapes of the kinetic curves and, as a consequence, the aggregation kinetic parameters (Figures 4d–f and S9), whereas the reduction in fibril length was comparable between trypsin‐treated (Figure S10) and untreated EVs (Figure 3). When used at the same particle concentration as untreated EVs, trypsinized EVs produced a saturating inhibitory effect. We therefore also examined a lower concentration range, where aggregation kinetic data, including half‐times, show that dose dependence was restored (Figure S11). Altogether this suggests that trypsinized EVs have a more potent inhibitory effect compared to their untreated counterparts. We excluded the possibility that residual trypsin would cause this enhanced inhibition by simply digesting Aβ(1–42) monomers by repeating the kinetic experiments using the retentate of an ultrafiltrated trypsin‐only sample. This had no effect on Aβ(1‐42) aggregation (Figure S12). Thus, it appears that EV surface proteins contribute to modulate Aβ(1‐42) aggregation and that their removal substantially enhances inhibition, possibly because Aβ(1‐42) species can more readily interact with EV membrane lipids.

### 2.3 EV‐associated proteoglycans have minor effect on Aβ(1‐42) aggregation

Proteolytic digestions of EV surface proteins also remove protein‐attached carbohydrates (glycosaminoglycans, GAGs). To explore whether EV cell‐surface carbohydrates are important in Aβ(1‐42) aggregation, we used two complementary experimental approaches. First, we compared the aggregation inhibitory effect of heparinase II treated and normal SH‐SY5Y derived EVs (Figure 5a,b). They had largely similar efficacy, but produced subtle differences to aggregation kinetic parameters, especially at the highest EV concentration tested (Figures 5c and S13a,b, two‐way ANOVA; *p* < 0.005). Since sugars have no specific absorption or emission properties it was not straightforward to quantify the extent of their removal. NTA‐based comparison of EV size revealed no consistent differences between heparinase‐treated and untreated EVs (Table S8), which is not unexpected given that other surface glycans, including chondroitin sulfate and glycans associated with glycoproteins and glycolipids, remain unaffected by heparinase treatment. We therefore complemented this approach by comparing EVs from two Chinese hamster ovary (CHO) cell lines with respectively normal (CHO‐K1) and deficient (CHO‐pgsA‐745) GAG expression (Esko et al., 1985). CHO‐pgsA‐745 cells lack xylosyltransferase which is responsible for the initial sugar transfer in GAG synthesis and this cell line therefore completely lacks protein‐bound sulfated sugars (Esko et al., 1985) resulting in a lower zeta potential (Amand et al., 2012). The EVs from the GAG‐deficient CHO‐pgsA‐745 cells were approximately 7 nm in diameter smaller than EVs from wildtype CHO‐K1 cells (Table S8), consistent with a contribution of surface‐associated GAGs to EV hydrodynamic size. Aggregation kinetics experiments showed that EVs from both these cell types slowed down Aβ(1‐42) aggregation (Figure 5d,e) and reduced fibril lengths (Figure S10), representing yet an example of EVs with inhibitory effect. The Aβ(1‐42) kinetic curves obtained in the presence of GAG‐deficient EVs had slightly longer *t*_half_, *t*_growth_, and *t*_lag_ (Figures 5f and S13c,d; two‐way ANOVA; *p* < 0.05). Altogether, this suggests that proteoglycans contribute to modulate EV‐mediated inhibition of Aβ(1‐42) aggregation, albeit to a much lesser extent than EV surface proteins.

**FIGURE 5:**
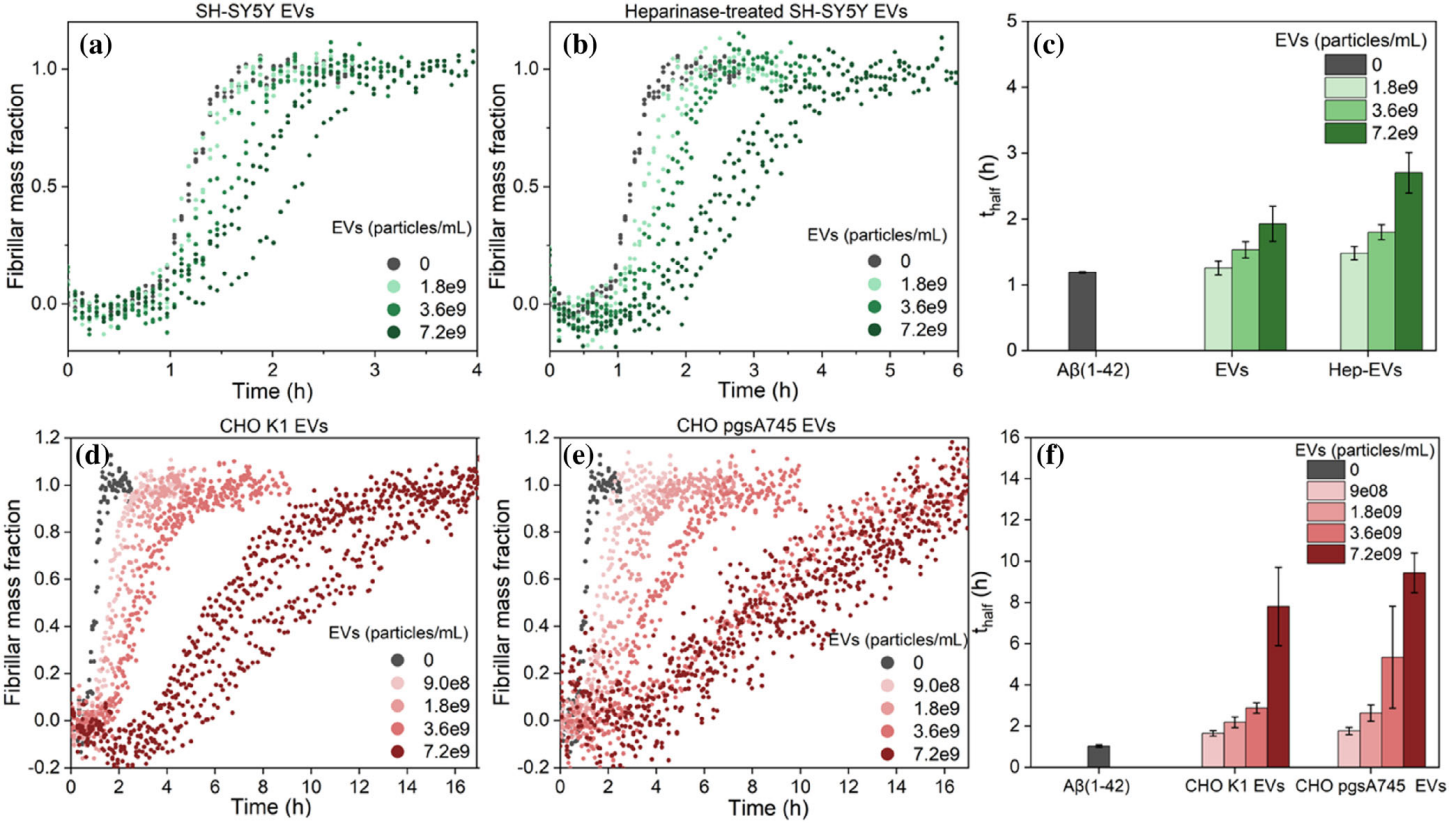
The effect of EV proteoglycans on Aβ(1‐42) aggregation kinetics by glycosaminoglycan modified vs non‐modified SH‐SY5Y and CHO EVs. (a and b) Normalized Aβ(1‐42) aggregation kinetic curves showing the effect SH‐SY5Y vs HSPG‐lacking SH‐SY5Y EVs. (c) Half‐times extracted from (a and b). (d and e) Normalized Aβ(1‐42) aggregation kinetic curves showing the effect of EVs from wildtype CHO‐K1 cells and pgsA‐745 mutant CHO cells. (f) Half‐times extracted from (d and e). Statistics: Two‐way ANOVA for factors EV type and concentration; see full ANOVA results in Tables S9–S11.

## 3 DISCUSSION

Despite that Aβ aggregation and senile plaque deposition are the most well‐recognized pathological features of Alzheimer's disease (AD), key mechanistic and molecular understanding of the drivers and propagators of this pathology remains missing. This presents a pressing knowledge gap and bottleneck in drug discovery. This study explores EVs as modulators of the Aβ(1‐42) aggregation pathway. The central conclusion of the work is that EVs, irrespective of the lineage, have highly similar effects slowing down Aβ(1‐42) aggregation. This points toward an aggregation‐reducing mechanism that is largely conferred by common EV properties rather than by specific EV ligands.

EVs have been implicated as versatile mediators of the pathology of AD (Da Conceicao et al., 2025; Gabrielli et al., 2022; Vingtdeux et al., 2012) and other neurodegenerative diseases (Hill, 2019) through actions involving, for example, transport of protein aggregates (Rajendran et al., 2006) or plaque clearance (Elia et al., 2019). EVs have also been reported to impact cognitive function (Wang & Yang, 2021). This study focuses directly on EV‐Aβ(1‐42) interactions, which may, for example, occur in the interstitial space of the brain, where animal studies indicate that EV concentrations increase with the emergence of plaque pathology (Pait et al., 2024). Our study shows that EVs from three different cell lines (MSC, THP‐1, and Jurkat) slow down Aβ(1‐42) aggregation. The kinetic profiles showed high similarity to those in a previous study of SH‐SY5Y and HEK293‐T derived EVs, in which seeded aggregation experiments demonstrated that the inhibitory effect was retained under elongation‐dominated conditions (Halipi et al., 2024). This, together with data fitting and the observation that the MSC, THP‐1, and Jurkat derived EVs also induced the formation of shorter Aβ(1‐42) fibrils, supports a shared mechanism of EV‐mediated inhibition of fibril elongation across EVs from different cell sources. Short fibrils, akin to oligomers, are diffusible and have been generally associated with neurotoxicity (Xue et al., 2009; Zhang et al., 2020). We have thus previously suggested that EVs have the capacity to alter the Aβ aggregation landscape in both protective (aggregation‐suppressive) and putatively detrimental ways.

The observation that EVs have generic rather than specific abilities to slow down Aβ(1‐42) aggregation is interesting because a more common notion in the EV field is that EVs (or at least a subset of EVs) rather have highly specialized functions that are tied to their exact biological compositions. Our data highlight that, in the context of protein aggregation, it is rather commonalities in biophysical and biochemical features that are important and a key focus of this study has therefore been to identify and understand the relative contribution of different biomolecular classes. Nevertheless, we observed modes of differences in the potency of EVs from different cell sources to inhibit Aβ(1‐42) aggregation, suggesting that differences in EV composition, including the surface proteome, glycan profile, and lipid composition, may modulate their interactions with monomeric and fibrillar Aβ(1‐42), thereby contributing to variations in inhibitory activity between EV types.

The EV surface is normally densely populated by both membrane‐incorporated and more loosely associated proteins. These proteins participate in targeting, cell interactions, cell uptake, and cell signaling (Wang et al., 2025), contributing to EV specialization and functionalization. Some are broadly shared across EV types and often linked to EV biogenesis and release, whereas other proteins are more specialized, reflecting their cellular origin and microenvironment (Amin et al., 2024). This study shows that generic (trypsin‐mediated) removal of the surface‐associated proteins on EVs enhances their inhibitory effect on Aβ(1‐42) aggregation. This could suggest that the lipid bilayer, which likely becomes more exposed when surface proteins are digested, has a particularly potent inhibitory effect and that EV proteins in fact act to modulate or even hinder inhibitory EV‐Aβ(1‐42) interactions. This could be due to steric hindrance or shielding constraints; notably, our study shows that trypsination leads to an approximate 4 nm thinning of the outer EV leaflet, supporting that EVs have a dense protein layer limiting direct access to their membrane lipids. Interestingly, a proteomic study on EVs isolated from interstitial fluid of live mice reported that EV concentrations increase, but their protein levels decrease with Aβ plaque formation (Pait et al., 2024). This could result in that brain‐derived EVs gradually lose their inhibitory potency with the progression of AD pathology and suggests that EVs may contribute to uphold extracellular proteostasis.

Following trypsin‐mediated protein removal, the EV surface can, to approximation, be considered lipid‐dominated. EV membranes are typically enriched in lipids such as cholesterol (Chol), sphingomyelin (SM), and phosphatidylserine (Ghadami & Dellinger, 2023; Skotland et al., 2020), but also various gangliosides (He et al., 2022). Brain‐relevant lipids such as Chol, glycolipids, and SM, which are, alongside phospholipids, common in EVs, have been studied by us and others in the context of Aβ(1‐42) aggregation and reported to either catalyze or inhibit Aβ(1‐42) fibril formation (Amaro et al., 2016; Calamai & Pavone, 2013; Habchi et al., 2018; Lindberg et al., 2017; Owen et al., 2018). Studies investigating the effects of mixed lipid membranes, which are arguably more relevant in the context of EVs, report a variety of outcomes, ranging from findings suggesting that more complex membranes balance opposing effects of individual lipids on Aβ(1‐42) aggregation (Sanguanini et al., 2020) to reports of either accelerated or inhibited aggregation depending on membrane composition (Banerjee et al., 2021; Baumann et al., 2023). We have recently shown that synthetic, protein‐free, lipid vesicles with SM/Chol/GM1‐content can both catalyze and inhibit Aβ(1‐42) aggregation depending on their exact lipid compositions (Sasanian et al., 2025). Furthermore, we showed that under lipid‐raft promoting compositions, the synthetic vesicles always inhibited Aβ(1‐42) aggregation. This is interesting given that EV membranes are generally reported to be more rigid than typical plasma cell membranes (Laulagnier et al., 2004; Parolini et al., 2009) and furthermore relevant because Aβ is known to bind to clusters of glycosphingolipids (Ariga et al., 2008; Yanagisawa et al., 1995), which are also enriched on EV surfaces (Yuyama et al., 2014). However, given the complexity of EV membranes and the fact that comprehensive lipidomic data on EVs remain limited, it is challenging to fully recapitulate their key features in simplified synthetic lipid models. Future studies combining EV lipidomic profiling with the extraction of EV lipids and their reconstitution into synthetic vesicles could help better establish how native EV lipid compositions contribute to Aβ(1‐42) self‐assembly.

Notably, the aggregation kinetics we observe for Aβ(1‐42) in the presence of trypsin‐treated EVs have a conspicuous extended lag phase, similar to what we report for GM1‐mediated inhibition (Sasanian et al., 2025) and explain by primary nucleation inhibition. This contrasts with the identification of EVs as inhibitors of fibril elongation in this and our previous (Halipi et al., 2024) work and suggests that EV surface proteins modulate not only the rate but also their mechanism of inhibition.

Lastly, this study also explores the possible contribution of protein‐attached EV glycans to Aβ(1‐42) aggregation showing that they have extremely modest effects. This is interesting given the reported roles of proteoglycans in Aβ uptake and accumulation (Sandwall et al., 2010; Wesen et al., 2018) and reports that soluble heparin can modulate Aβ self‐assembly (Radko et al., 2018). Glycans are also considered as generally important for the function of EVs (Williams et al., 2018).

In conclusion, this study shows that EVs from a variety of cell sources have common and potent abilities to inhibit Aβ(1‐42) aggregation, expanding our previous findings (Halipi et al., 2024). Moreover, we show that EV surface proteins, and to a lesser extent carbohydrates, are important modulators of the aggregation‐inhibitory effect that we propose originates from Aβ(1‐42) interactions with the EV lipid interface. Altogether, this work provides a more detailed understanding for the role of EVs in AD pathology and pinpoints them as important aggregation regulators and putative contributors to protein homeostasis, acting in the interstitial space.

## 4 MATERIALS AND METHODS

### 4.1 Cell culture and cell lines

SH‐SY5Y cells were cultured in 1:1 medium of MEM + GlutaMAX and F‐12 Nut Mix (Gibco, USA) supplemented with 10% FBS and 1% non‐essential amino acids (Gibco, USA). CHO cells were cultured in DMEM (Gibco, USA) supplemented with 10% FBS. Cell line authentication based on genotyping was performed according to ANSI/ATCC standard ASN‐0002 (Eurofins Genomics) for SH‐SY5Y cells. SH‐SY5Y and CHO cells were cultured in T75 flasks (Thermo Scientific) at 37°C and 5% CO_2_ until 70% confluence, after which cell medium (CM) was exchanged to 1:1 medium of MEM + GlutaMAX and F‐12 Nut Mix without FBS for SH‐SY5Y and DMEM without FBS for CHO cells for 48 h. CM was collected for EV isolation.

Umbilical cord‐blood derived mesenchymal stromal cells (MSC) were cultured in MEM‐α modification medium (containing l‐glutamine; Thermo Fisher Scientific) supplemented with basic fibroblast growth factor (5 ng/mL; Sigma–Aldrich) and 10% FBS and 1X antibiotic‐antimycotic (Thermo Fisher Scientific) as described previously (Hagey et al., 2023). THP‐1 and Jurkat cells were cultured in RPMI‐1640 medium containing GlutaMAX‐I and 25 mM HEPES (Invitrogen) supplemented with 10% FBS and 1× antibiotic‐antimycotic. Cells were cultured at 37°C and 5% CO_2_ and regularly tested for the presence of mycoplasma. The CM was changed to optiMEM (Invitrogen) without FBS for 48 h before collection of CM for EV isolation.

### 4.2 EV isolation

EVs were collected as previously described (Halipi et al., 2024). CM was centrifuged at low speed (2000 g) for 20 min to remove larger particles and cell debris and subsequently filtered through a 0.22 μm pore size (VWR) membrane. This was followed by ultrafiltration with Amicon Ultra‐15 10 kDa (Millipore) spin filters at 5000 *g* for 2 h. The collector tube was emptied, and Dulbecco's phosphate‐buffered saline (DPBS) (ThermoFisher Scientific) was added prior to a second centrifugation at the same speed. SH‐SY5Y EVs were stored in DPBS at 4°C and used within 2 days for all experiments. All samples were stored on ice during the isolation process. Isolation of EVs from umbilical cord‐blood derived mesenchymal stromal cells (MSC), THP‐1 and Jurkat cells was performed by centrifuging CM at low speed (500 *g*) for 5 min followed by a 2000 *g* spin for 10 min to remove larger particles and cell debris, after which the pre‐cleared cell culture supernatant was filtered through a 0.22 μm bottle top vacuum filter (Corning). EVs were first prepared by tangential flow filtration (TFF) using a KR2i TFF system (SpectrumLabs) equipped with modified polyethersulfone (mPES) hollow fiber filters with 300 kDa membrane pore size (MidiKros, 370 cm^2^ surface area, SpectrumLabs) at a flow rate of 100 mL/min (transmembrane pressure at 3.0 psi and shear rate at 3700 s^−1^) as described previously (Corso et al., 2017). Amicon Ultra‐0.5 10 kDa MWCO spin‐filters (Millipore) were used to concentrate the sample to a final volume of 100 μL. These EVs were stored in DPBS buffer containing 0.2% albumin, 25 mM HEPES and 25 mM Trehalose (PBS‐HAT) (Gorgens et al., 2022). The EVs were stored at −80°C upon delivery and thawed on ice directly prior to all experiments.

### 4.3 EV trypsin treatment

EVs were treated with trypsin (Gibco) at a concentration of 1.25 mg/mL at 37°C for 30 min. DPBS was added to control EV samples not subjected to trypsin treatment, and these were also incubated at 37°C for 30 min. After the incubation, samples were diluted in DPBS, introduced to Amicon Ultra‐15 30 kDa (Millipore) spin filters, and centrifuged at 6400 rpm with several DPBS washes to remove trypsin and peptide fragments. EVs were collected, stored at 4°C, and used within 2 days for all experiments.

### 4.4 Heparinase treatment

We utilized a heparinase II (25 Units/mL) from Flavobacterium heparinum (Sigma–Aldrich), which was diluted in DPBS to a concentration of 2.5 U/mL, aliquoted and stored at −20°C until further use. SH‐SY5Y EVs were treated with 5, 10, or 20 mU/mL for 2 h or with 20 mU/mL for 3, 6, and 24 h. Only data from the 20mU/mL treatment for 24 h are shown. EV samples treated with heparinase were kept at 4°C and used within 2 days for aggregation experiments. No further steps were taken to remove the heparinase or any heparan sulfate (HS) fragments.

### 4.5 Nanoparticle tracking analysis (NTA)

Mean size (diameter) and concentration of EVs were determined by NTA using a NanoSight LM14 instrument with NTA 3.3 software (Malvern Instruments). Five consecutive 60 s videos were recorded per sample in light scatter mode, screen gain 5.0, and camera setting of 14. All EV samples were diluted in 0.22 μm filtered DPBS prior to NTA measurements. Software settings were kept constant for all measurements to obtain comparable results. A detection threshold of 2 for SH‐SY5Y or 3 for umbilical cord‐blood MSC, THP‐1, Jurkat EVs, and CHO EVs was used, respectively.

### 4.6 Fluorescence spectroscopy measurements

Extracellular vesicles were isolated from cultured cells and subjected to trypsin treatment as described above. Intrinsic fluorescence from aromatic amino acids (primarily tryptophan and tyrosine) was recorded for untreated and trypsin‐treated EV samples using a Cary Eclipse fluorescence spectrophotometer (Agilent). Samples were measured in a 10 mm path length quartz cuvette. Emission spectra were collected from 300 to 500 nm with an excitation wavelength of 280 nm. Both excitation and emission slit widths were set to 5 nm. Spectra were acquired at a scan rate of 600 nm/min using a photomultiplier tube (PMT) voltage of 600 V.

### 4.7 Aβ(1‐42) expression and purification

Recombinant Aβ(1‐42) was expressed overnight in E. coli (BL21) as a fusion protein with an NT* solubility tag (Abelein et al., 2020). In brief, bacterial cells were centrifuged and dissolved in 20 mM Tris–HCl, 8 M Urea, pH 8.0 and frozen at −20°C until further use. For Aβ(1‐42)‐NT purification, cells were sonicated, centrifuged and filtered prior to loading to a HisPrep FF 16/10 column (GE Healthcare). Aβ(1‐42)‐NT was eluted in a 20 mM Tris–HCl, 8 M Urea, 300 mM Imidazole, pH 8.0 buffer, and cleaved with a TEV (tobacco etch virus) protease during dialysis in a 20 mM Tris–HCl, pH 8.0 buffer with 0.5 mM EDTA and 1.5 mM DTT at 4°C overnight. The cleaved Aβ(1‐42) was purified on a HiLoad 16/600 Superdex 30 pg. (GE Healthcare) size exclusion column with a 20 mM sodium phosphate, pH 8.0 buffer. The Aβ(1‐42) peptide was aliquoted, snap frozen, lyophilized and stored in −20°C until further use. Prior to all kinetics experiments and AFM imaging, lyophilized Aβ(1‐42) was dissolved in 6 M guanidine hydrochloride for 20 min on ice before purification by size exclusion chromatography in a 20 mM sodium phosphate buffer, pH 8.0 using a 10/300 Superdex 75 column (GE Healthcare). Monomeric Aβ(1‐42) was eluted as a single peak at approximately 14 mL and the concentration was determined from the integrated peak area in the chromatogram (*ε*_280nm_ = 1280/M/cm), as previously reported by us (Halipi et al., 2024).

### 4.8 Thioflavin‐T (ThT) monitored aggregation kinetics

Samples containing 2 μM of monomeric Aβ(1‐42) in 20 mM phosphate buffer, pH 8.0 (prepared as described above), 5 μM ThT, and indicated concentrations of EVs (particles/mL) were prepared on ice and distributed in triplicate to non‐binding, transparent bottom, black half‐area 96‐well microplates (Corning) sealed with adhesive film to prevent evaporation. ThT emission was measured with bottom optics in a FluoStar OPTIMA fluorescence plate reader (BMG Labtech) and a 440 ± 10 nm and a 485 ± 10 nm bandpass filter for excitation and emission respectively. All ThT measurements were performed at 37°C and quiescent conditions. The aggregation kinetic parameters half time (*t*_half_), lag time (*t*_lag_), and growth time (*t*_growth_) were extracted from the ThT fluorescence aggregation curves. *t*_half_ denotes the reaction time to reach 50% of the total fibrillar mass formation, *t*_lag_ corresponds to the reaction time at which 10% of the final fibril content is attained, and *t*_growth_ denotes the reaction time needed to increase the fibril content from 10% to 90% of the end‐point maximum.

### 4.9 Analysis and modeling of kinetic data

The acquired kinetic data were analyzed to determine the dominant mechanism of aggregation and extract rate constants. A multi‐step secondary nucleation dominated model (Cohen et al., 2013) on the web‐based tool AmyloFit (Meisl, Kirkegaard, et al., 2016) was used to fit the experimental kinetic data. Experimental data for Aβ(1‐42) in buffer was fitted, enabling estimation of rate constants for elongation and primary nucleation or elongation and secondary nucleation; *k*_+_ *k*_*n*_ and *k*_+_ *k*_2_, respectively. KM was determined and kept constant for further analysis. These values were used as initial guesses when analyzing Aβ(1‐42) aggregation kinetics data in the presence of EVs. The same model, with initial guesses, was used to fit Aβ(1‐42) aggregation data in the presence of EVs, yielding combined rate constants for elongation and primary nucleation or elongation and secondary nucleation; *k*_+_ *k*_*n*_ and *k*_+_ *k*_2_, respectively. The goodness of fits was evaluated based on mean residual errors.

### 4.10 Atomic force microscopy

Samples for atomic force microscopy (AFM) were prepared by depositing 10 μL of sample onto a freshly cleaved mica surface functionalized with 10 μL (3‐aminopropyl)triethoxysilane for 30 s, washed with Milli Q water, and dried with nitrogen gas. Samples were incubated on the mica for 10 min to let fibrils attach and then the mica was washed with MQ followed by drying with nitrogen gas. All samples were stored in closed containers until imaging began. Recording of AFM images was performed using an NTEGRA system with a gold‐covered crystal cantilever (NT‐MDT, NSG01, force constant ~ 5.1 N/m, resonance frequency ~150 Hz). The 10 × 10 μm images with 256 × 256 pixels were recorded for all samples and images were analyzed using Gwyddion software by planar subtraction, polynomial background subtraction, correction of horizontal aberrations and minimum value shift to zero. One hundred Aβ(1‐42) fibrils per sample were measured for fibril length and height based on 10–11 AFM images. The data were analyzed by performing a one‐way ANOVA Post‐hoc Bonferroni‐corrected means comparison tests [OriginPro 2020 software (OriginLab)].

## AUTHOR CONTRIBUTIONS

**Vesa Halipi:** Conceptualization; investigation; writing – original draft; methodology; validation; visualization; writing – review and editing; formal analysis; data curation. **Georgia Daoutsali:** Investigation; methodology; validation; formal analysis; data curation. **Fritjof Havemeister:** Formal analysis; data curation; investigation. **Viktoria de Carvalho:** Investigation. **André Görgens:** Writing – review and editing; resources. **Samir El Andaloussi:** Resources. **Elin K. Esbjörner:** Writing – original draft; funding acquisition; investigation; conceptualization; visualization; writing – review and editing; validation; supervision; resources; project administration; formal analysis.

## Supporting information


**Figure S1:** Size and concentration distributions of EVs. Nanoparticle Tracking Analysis (NTA) data showing the size and concentration distribution of cord‐blood derived MSC, THP‐1 and Jurkat EVs. Mean EV diameter ± standard deviations for each EV type are given in the legend.
**Figure S2:** Comparison of *t*_half_ for Aβ(1‐42) aggregation in presence of MSC, THP‐1 and Jurkat EVs vs. SH‐SY5Y and HEK293‐T EVs. Relative reaction half‐times (*t*_half_) for Aβ(1‐42) aggregation in presence of EVs, expressed relative to *t*_half_ of the corresponding Aβ(1‐42)‐only control in each experiment. Data were taken from Figure 2d and *t*_half_ of Aβ(1‐42) aggregation in presence of HEK293‐T and SH‐SY5Y EVs, data derived from our previous study.^1^
**Figure S3:** Kinetic modeling of experimental Aβ(1‐42) aggregation kinetics data using AmyloFit. (a‐c) Normalized aggregation kinetics data of 2 μM Aβ(1‐42) in absence or presence of indicated EVs, corresponding to the data in Figure 2a–c in the main text. Dotted lines represent experimental data and solid lines are fitted using the web‐based tool AmyloFit and a multi‐step secondary nucleation aggregation model. The combined rate constants for elongation and primary nucleation (*k*_+_ *k*_*n*_) was set as a free parameter, as described in Section 4.
**Figure S4:** Atomic force microscopy (AFM) of Aβ(1‐42) fibrils. Representative AFM images of Aβ(1‐42) fibrils formed in absence and presence of the indicated EVs (at an EV concentration of 7.2e09 particles/mL). Scale bar = 2 μm.
**Figure S5:** AFM image of SH‐SY5Y EVs. Representative AFM image of SH‐SY5Y EVs (1e11 particles/mL) showing the existence of a heterogeneous background distinct from mica with only Aβ(1‐42) present. Scale bar = 4 μm.
**Figure S6:** Analysis of cross‐sectional heights of Aβ(1‐42). Cross‐sectional heights analysis of Aβ(1‐42) fibrils formed in absence or presence of MSC, THP‐1 and Jurkat EVs based on AFM images (see Section 4) (mean ± standard deviation, *n* = 100 fibrils from 11 images for the Aβ(1‐42) + THP‐1 EVs condition and from 10 images for the other conditions, *** denotes *p* < 0.001, by one‐way ANOVA and Post‐hoc Bonferroni‐corrected means comparison tests). The EV concentration was 7.2e9 particles/mL in all conditions containing EVs. Box plots represent the interquartile range (25–75th percentile), mean values are presented as squares, median values as horizontal lines. Whiskers indicate values within 1.5 × the interquartile range and black diamonds show outliers.
**Figure S7:** EV concentration before and after trypsin‐treatment. The distribution plots show the mean concentration of EVs ± SD of untreated vs. trypsin‐treated EVs (*n* = 9). Two Sample *T*‐test revealed that the concentration does not change significantly after trypsin‐treatment (*p* > 0.2).
**Figure S8:** Characterization of surface protein digestion after EV trypsin‐treatment. Tryptophan (and tyrosine) fluorescence of buffer (DPBS) and trypsin‐treated buffer after ultrafiltration, as described in Figure 4 and in Section 4.
**Figure S9:** Reaction parameters derived from Aβ(1‐42) aggregation kinetics in presence of untreated or trypsin‐treated EVs. Lag‐times (a) and growth‐times (b) of Aβ(1‐42) aggregation in absence or presence of SHSY5Y EVs and trypsin‐treated SHSY5Y EVs, derived from the data in Figure 4a,b.
**Figure S10:** Fibril lengths. Analysis of fibril lengths, expressed relative to the length of the Aβ(1‐42)‐only control fibrils, based on AFM images of Aβ(1‐42) fibrils (mean ± standard deviation, *n* = 100 fibrils for the Aβ(1‐42), Aβ(1‐42) + CHO K1 EVs, Aβ(1‐42) + CHO pgsA 745 EVs and *n* = 80 for Aβ(1‐42) + Tryp SH‐SY5Y EVs).
**Figure S11:** Aβ(1‐42) aggregation kinetics in the presence of trypsin‐treated SH‐SY5Y EVs. (a) Normalized ThT fluorescence data showing Aβ(1‐42) aggregation (2 μM) in absence or presence of trypsin‐treated EVs across an extended range of EV concentrations including those used in Figure 4d,e and additional samples covering lower concentrations, *n* = 3. (b) Half‐times of Aβ(1‐42) aggregation (2 μM) in absence or presence of trypsin‐treated EVs extracted from (a).
**Figure S12:** Aβ(1‐42) aggregation kinetics in the presence of trypsin‐control (DPBS). Normalized aggregation kinetics data of 2 μM Aβ(1‐42) in untreated or DPBS buffer subjected to the same trypsin‐treatment and trypsin‐removal protocol as the EV samples (see Section 4 for details), but in the absence of EVs (“trypsin‐treated buffer”). The indicated values (9.0e8–7.2e9) correspond to the EV concentrations (particles/mL), and thus volumes, used in Figure 4.
**Figure S13:** Reaction parameters derived from Aβ(1‐42) aggregation kinetics. Lag‐times (a) and growth‐times (b) of Aβ(1‐42) aggregation in absence or presence of untreated or heparinase‐treated SHSY5Y EVs. Lag‐times (c) and growth‐times (d) of Aβ(1‐42) aggregation in absence or presence of CHO K1 or CHO pgsA‐745 EVs. The reaction parameters are derived from aggregation curves in Figure 5.
**Table S1:** Overall two‐way ANOVA analysis of EV type and concentration on *t*_half_ of Aβ(1‐42) aggregation kinetics. Summary of the two‑way ANOVA evaluating the effects of EV type (MSC, THP‐1 and Jurkat) and EV concentration on the *t*_half_ of Aβ(1‑42) aggregation. The table lists degrees of freedom, sums of squares, mean squares, *F*‑values, and *p*‑values for each factor, their interaction, and the overall model.
**Table S2:** Overall two‐way ANOVA analysis of EV type and concentration on *t*_lag_ of Aβ(1‐42) aggregation kinetics. Summary of the two‑way ANOVA evaluating the effects of EV type (MSC, THP‐1 and Jurkat) and EV concentration on the *t*_lag_ of Aβ(1‑42) aggregation. The table lists degrees of freedom, sums of squares, mean squares, *F*‑values, and *p*‑values for each factor, their interaction, and the overall model.
**Table S3:** Overall two‐way ANOVA analysis of EV type and concentration on *t*_growth_ of Aβ(1‐42) aggregation kinetics. Summary of the two‑way ANOVA evaluating the effects of EV type (MSC, THP‐1 and Jurkat) and EV concentration on the *t*_growth_ of Aβ(1‑42) aggregation. The table lists degrees of freedom, sums of squares, mean squares, *F*‑values, and *p*‑values for each factor, their interaction, and the overall model.
**Table S4:** Fitted kinetics parameters for Aβ(1‐42) in presence of cord‐blood derived MSC EVs. Calculated kinetics parameters *k*_+_ *k*_*n*_ (combined rate constants for elongation and primary nucleation), *k*_+_ *k*_2_ (combined rate constants for elongation and secondary nucleation) using a multi‐step secondary nucleation on the web‐based tool AmyloFit. Corresponding experimental data and fittings are seen in Figure 2a. *K*_M_ (Michaelis constant) was determined for Aβ(1‐42) aggregation in absence of EVs and was kept as a global constant for all further analyses.
**Table S5:** Fitted kinetics parameters for Aβ(1‐42) in presence of THP‐1 EVs. Calculated kinetics parameters *k*_+_ *k*_*n*_ (combined rate constants for elongation and primary nucleation), *k*_+_ *k*_2_ (combined rate constants for elongation and secondary nucleation) using a multi‐step secondary nucleation on the web‐based tool AmyloFit. Corresponding experimental data and fittings are seen in Figure 2b. *K*_M_ (Michaelis constant) was determined for Aβ(1‐42) aggregation in absence of EVs and was kept as a global constant for all further analyses.
**Table S6:** Fitted kinetics parameters for Aβ(1‐42) in presence of Jurkat EVs. Calculated kinetics parameters k_+_k_n_ (combined rate constants for elongation and primary nucleation), k_+_k_2_ (combined rate constants for elongation and secondary nucleation) using a multi‐step secondary nucleation on the web‐based tool AmyloFit. Corresponding experimental data and fittings are seen in Figure 2c. *K*_M_ (Michaelis constant) was determined for Aβ(1‐42) aggregation in absence of EVs and was kept as a global constant for all further analyses.
**Table S7:** Mean diameter of untreated or trypsin‐treated SH‐SY5Y EVs. The diameter size in each group (*n* = 9) was determined using NTA as described in Section 4.
**Table S8:** Hydrodynamic diameters of heparinase treated (hep‐treated) SH‐SY5Y EVs, CHO‐K1 EVs and GAG‐deficient CHO pgsA745 EVs. Data were acquired using NTA. *n* = 1 for SH‐SY5Y EVs and *n* = 2 for CHO EVs.
**Table S9:** Overall two‐way ANOVA analysis of EV type and concentration on *t*_half_ of Aβ(1‐42) aggregation kinetics.
**Table S10:** Overall two‐way ANOVA analysis of EV type and concentration on *t*_lag_ of Aβ(1‐42) aggregation kinetics.
**Table S11:** Overall two‐way ANOVA analysis of EV type and concentration on *t*_growth_ of Aβ(1‐42) aggregation kinetics.

## Data Availability

All data needed to evaluate the conclusions in the paper are present in the paper and/or the Supplementary Information. Raw data supporting the findings of this manuscript are available from the corresponding author upon request.
