# Supplementary material for: Surface proteins modulate extracellular vesicle mediated inhibition of Aβ(1‐42) aggregation
Source: Protein Sci. 2026 Nov 5;35(12):e70849. doi: 10.1002/pro.70849 (PMC13740363; doi:10.1002/pro.70849)
Supplement: Supplementary file 1 [file PRO-35-e70849-s001.docx]

**Supplementary information**

**to**

**Surface proteins modulate extracellular vesicle mediated inhibition of Aβ(1-42) aggregation**

Vesa Halipi^1^, Georgia Daoutsali^1^, Fritjof Havemeister^1^, Viktoria de Carvalho^1^, André Görgens^2^, Samir El Andaloussi^2^, Elin K. Esbjörner^1^*

^1^Division of Chemical Biology, Department of Life Sciences, Chalmers University of Technology, Kemivägen 10, 412 96 Gothenburg, Sweden

^2^Division of Biomolecular and Cellular Medicine, Department of Laboratory Medicine, Karolinska Institute, Stockholm 17177, Sweden

Corresponding author: Elin K. Esbjörner; [eline@chalmers.se](mailto:eline@chalmers.se)

**Figure S1. Size and concentration distributions of EVs.** Nanoparticle Tracking Analysis (NTA) data showing the size and concentration distribution of cord-blood derived MSC, THP-1 and Jurkat EVs. Mean EV diameter ± standard deviations for each EV type are given in the legend.

**Table S1.** **Overall two-way ANOVA analysis of EV type and concentration on t_half_ of** **Aβ(1-42) aggregation kinetics.** Summary of the two‑way ANOVA evaluating the effects of EV type (MSC, THP-1 and Jurkat) and EV concentration on the t_half_ of Aβ(1‑42) aggregation. The table lists degrees of freedom, sums of squares, mean squares, F‑values, and p‑values for each factor, their interaction, and the overall model.

|  | Degrees of Freedom | Sum of Squares | Mean Square | F value | *p*-value |
| --- | --- | --- | --- | --- | --- |
| EV type | 2 | 0.61037 | 0.30519 | 8.68682 | 0.00229 |
| Concentration | 2 | 4.24516 | 2.12258 | 60.41711 | <0.0001 |
| Interaction | 4 | 0.16223 | 0.04056 | 1.15444 | 0.36336 |
| Model | 8 | 5.01776 | 0.62722 | 17.8532 | <0.0001 |
| Residual (Error) | 18 | 0.63238 | 0.03513 |  |  |
| Corrected Total | 26 | 5.65014 |  |  |  |

**Table S2.** **Overall two-way ANOVA analysis of EV type and concentration on t_lag_ of** **Aβ(1-42) aggregation kinetics.** Summary of the two‑way ANOVA evaluating the effects of EV type (MSC, THP-1 and Jurkat) and EV concentration on the t_lag_ of Aβ(1‑42) aggregation. The table lists degrees of freedom, sums of squares, mean squares, F‑values, and p‑values for each factor, their interaction, and the overall model.

|  | Degrees of Freedom | Sum of Squares | Mean Square | F value | *p*-value |
| --- | --- | --- | --- | --- | --- |
| EV type | 2 | 0.58231 | 0.29115 | 7.181 | 0.00509 |
| Concentration | 2 | 2.0378 | 1.0189 | 25.12998 | <0.0001 |
| Interaction | 4 | 0.1372 | 0.0343 | 0.846 | 0.51431 |
| Model | 8 | 2.75731 | 0.34466 | 8.50075 | <0.0001 |
| Residual (Error) | 18 | 0.72981 | 0.04055 |  |  |
| Corrected Total | 26 | 3.48713 |  |  |  |

**Table S3.** **Overall two-way ANOVA analysis of EV type and concentration on t_growth_ of** **Aβ(1-42) aggregation kinetics.** Summary of the two‑way ANOVA evaluating the effects of EV type (MSC, THP-1 and Jurkat) and EV concentration on the t_growth_ of Aβ(1‑42) aggregation. The table lists degrees of freedom, sums of squares, mean squares, F‑values, and p‑values for each factor, their interaction, and the overall model.

|  | Degrees of Freedom | Sum of Squares | Mean Square | F value | *p*-value |
| --- | --- | --- | --- | --- | --- |
| EV type | 2 | 0.47966 | 0.23983 | 6.02358 | 0.00994 |
| Concentration | 2 | 2.02503 | 1.01251 | 25.43059 | <0.0001 |
| Interaction | 4 | 0.60627 | 0.15157 | 3.80684 | 0.02059 |
| Model | 8 | 3.11096 | 0.38887 | 9.76696 | <0.0001 |
| Residual (Error) | 18 | 0.71667 | 0.03981 |  |  |
| Corrected Total | 26 | 3.82762 |  |  |  |

**Fig. S2 Comparison of t_half_ for Aβ(1-42) aggregation in presence of MSC, THP-1 and Jurkat EVs vs SH-SY5Y EVs.** Relative reaction half-times (t_half_) for Aβ(1-42) aggregation in presence of MSC, THP-1 and Jurkat EVs, expressed relative to t_half_ of the corresponding Aβ(1-42)-only control in each experiment. Data were taken from Fig. 2d and t_half_ of Aβ(1-42) aggregation in presence of HEK293-T and SH-SY5Y EVs, data derived from our previous study (Halipi et al. 2024).

**Figure S3. Kinetic modelling of experimental Aβ(1-42) aggregation kinetics** **data using AmyloFit.** (**a**-**c**) Normalized aggregation kinetics data of 2 µM Aβ(1-42) in absence or presence of indicated EVs, corresponding to the data in Fig. 2a-c in the main text. Dotted lines represent experimental data and solid lines are fitted using the web-based tool AmyloFit and a multi-step secondary nucleation aggregation model. The combined rate constants for elongation and primary nucleation (k_+_k_n_) was set as a free parameter, as described in Methods.

**Table S4. Fitted kinetics parameters for Aβ(1-42) in presence of cord-blood derived MSC EVs.** Calculated kinetics parameters k_+_k_n_ (combined rate constants for elongation and primary nucleation), k_+_k_2_ (combined rate constants for elongation and secondary nucleation) using a multi-step secondary nucleation on the web-based tool AmyloFit. Corresponding experimental data and fittings are seen in Fig. 2a. K_M_ (Michaelis constant) was determined for Aβ(1-42) aggregation in absence of EVs and was kept as a global constant for all further analyses.

|  | Umbilical cord-blood derived MSC EVs (particles/mL) | | | | Aβ(1-42) only |
| --- | --- | --- | --- | --- | --- |
|  | 1.8e9 | 3.6e9 | 7.2e9 | Mean residual error |  |
| k_+_k_n_ (M^-nc^h^-2^) | 2.47e7 | 1.77e6 | 4.56e4 | 0.00717 | 4.86e9 |
| k_+_k_2_ (M^-n2-1^h^-2^) | 2.56e18 | 1.57e18 | 7.87e17 | 0.00485 | 7.11e18 |
| k_+_k_n_, k_+_k_2_ | 2.33e9, 3.62e18 | 4.01e8, 3.96e18 | 1.18e9, 1.58e18 | 0.00333 | 4.86e9, 7.11e18 |
| K_M_ (M^n2^) |  |  |  |  | 2.86e-12 |

**Table S5. Fitted kinetics parameters for Aβ(1-42) in presence of THP-1 EVs.** Calculated kinetics parameters k_+_k_n_ (combined rate constants for elongation and primary nucleation), k_+_k_2_ (combined rate constants for elongation and secondary nucleation) using a multi-step secondary nucleation on the web-based tool AmyloFit. Corresponding experimental data and fittings are seen in Fig. 2b. K_M_ (Michaelis constant) was determined for Aβ(1-42) aggregation in absence of EVs and was kept as a global constant for all further analyses.

|  | THP-1 EVs (particles/mL) | | | | Aβ(1-42) only |
| --- | --- | --- | --- | --- | --- |
|  | 1.8e9 | 3.6e9 | 7.2e9 | Mean residual error |  |
| k_+_k_n_ (M^-nc^h^-2^) | 2.21e6 | 4.93e4 | 2.78e3 | 0.00816 | 4.86e9 |
| k_+_k_2_ (M^-n2-1^h^-2^) | 1.11e18 | 5.71e17 | 3.78e17 | 0.00649 | 7.11e18 |
| k_+_k_n_, k_+_k_2_ | 1.67e8, 3.19e18 | 8.22e8, 1.16e18 | 3.32e8, 1.08e18 | 0.00352 | 4.86e9, 7.11e18 |
| K_M_ (M^n2^) |  |  |  |  | 2.86e-12 |

**Table S6. Fitted kinetics parameters for Aβ(1-42) in presence of Jurkat EVs.** Calculated kinetics parameters k_+_k_n_ (combined rate constants for elongation and primary nucleation), k_+_k_2_ (combined rate constants for elongation and secondary nucleation) using a multi-step secondary nucleation on the web-based tool AmyloFit. Corresponding experimental data and fittings are seen in Fig. 2c. K_M_ (Michaelis constant) was determined for Aβ(1-42) aggregation in absence of EVs and was kept as a global constant for all further analyses.

|  | Jurkat EVs (particles/mL) | | | | Aβ(1-42) only |
| --- | --- | --- | --- | --- | --- |
|  | 1.8e9 | 3.6e9 | 7.2e9 | Mean residual error |  |
| k_+_k_n_ (M^-nc^h^-2^) | 2.81e6 | 8.84e5 | 3.70e3 | 0.00963 | 4.86e9 |
| k_+_k_2_ (M^-n2-1^h^-2^) | 1.16e18 | 9.53e17 | 3.94e17 | 0.00923 | 7.11e18 |
| k_+_k_n_, k_+_k_2_ | 1.55e8, 3.37e18 | 1.97e8, 2.66e18 | 5.86e8, 9.38e17 | 0.00596 | 4.86e9, 7.11e18 |
| K_M_ (M^n2^) |  |  |  |  | 2.86e-12 |

**Fig. S4. Atomic force microscopy (AFM) of Aβ(1-42) fibrils.** Representative AFM images of Aβ(1-42) fibrils formed in absence and presence of the indicated EVs (at an EV concentration of 7.2e09 particles/mL). Scale bar = 2 µm.

**Fig. S5. AFM image of SH-SY5Y EVs.** Representative AFM image of SH-SY5Y EVs (1e11 particles/mL) showing the existence of a heterogeneous background distinct from mica with only Aβ(1-42) present. Scale bar = 4 µm.

**Fig. S6.** **Analysis of cross-sectional heights of Aβ(1-42).** Cross-sectional heights analysis of Aβ(1-42) fibrils formed in absence or presence of MSC, THP-1 and Jurkat EVs based on AFM images (see Methods ) (mean ± standard deviation, n = 100 fibrils from 11 images for the Aβ(1-42) + THP-1 EVs condition and from 10 images for the other conditions, *** denotes *p* < 0.001, by one-way ANOVA and Post-hoc Bonferroni-corrected means comparison tests). The EV concentration was 7.2e9 particles/mL in all conditions containing EVs. Box plots represent the interquartile range (25-75th percentile), mean values are presented as squares, median values as horizontal lines. Whiskers indicate values within 1.5 x the interquartile range and black diamonds show outliers.

**Table S7. Mean diameter of untreated or trypsin-treated SH-SY5Y EVs.** The diameter size in each group (n = 9) was determined using NTA as described in Methods.

|  | **Size (nm)** | |
| --- | --- | --- |
|  | **Untreated EVs** | **Trypsin-treated EVs** |
| **1.** | 63.7 | 55.2 |
| **2.** | 65.8 | 64.0 |
| **3.** | 70.4 | 68.2 |
| **4.** | 79.2 | 65.1 |
| **5.** | 75.4 | 71.6 |
| **6.** | 87.3 | 64.8 |
| **7.** | 77.0 | 61.8 |
| **8.** | 74.1 | 74.2 |
| **9.** | 74.7 | 73.0 |

**Fig. S7. EV concentration before and after trypsin-treatment.** The distribution plots show the mean concentration of EVs ± SD of untreated vs trypsin-treated EVs (*n* = 9). Two Sample T-test revealed that the concentration does not change significantly after trypsin-treatment (*p* > 0.2).

**Table. S8. Hydrodynamic diameters of heparinase treated (hep-treated) SH-SY5Y EVs, CHO-K1 EVs and GAG-deficient CHO pgsA745 EVs.** Data were acquired using NTA. n = 1 for SH-SY5Y EVs and n = 2 for CHO EVs.

| EV type | EV diameter (nm) |
| --- | --- |
| SHSY5Y (untreated) | 86.4 |
| 3h hep-treated SHSY5Y | 98.9 |
| 6h hep-treated SHSY5Y | 94.7 |
| 24h hep-treated SHSY5Y | 88.4 |
| CHO K1 | 90.3 |
| CHO pgsA745 | 83.1 |

**Figure S8. Characterization of surface protein digestion after EV trypsin-treatment.** Tryptophan (and tyrosine) fluorescence of buffer (DPBS) and trypsin-treated buffer after ultrafiltration, as described in Fig. 4 and in Methods.

**Figure S9.** **Reaction parameters derived from** **Aβ(1-42) aggregation kinetics in presence of untreated or trypsin-treated EVs.** Lag-times (**a**) and growth-times (**b**) of Aβ(1-42) aggregation in absence or presence of SHSY5Y EVs and trypsin-treated SHSY5Y EVs, derived from the data in Fig. 4a-b.

**Fig. S10. Fibril lengths.** Analysis of fibril lengths, expressed relative to the length of the Aβ(1-42)-only control fibrils, based on AFM images of Aβ(1-42) fibrils (mean ± standard deviation, n = 100 fibrils for the Aβ(1-42), Aβ(1-42) + CHO K1 EVs, Aβ(1-42) + CHO pgsA 745 EVs and n = 80 for Aβ(1-42) + Tryp SH-SY5Y EVs.

**Figure S11. Aβ(1-42) aggregation kinetics in the presence of trypsin-treated SH-SY5Y EVs.** (**a**) Normalized ThT fluorescence data showing Aβ(1-42) aggregation (2 µM) in absence or presence of trypsin-treated EVs across an extended range of EV concentrations including those used in Fig. 5d-e and additional samples covering lower concentrations, n=3. (**b**) Half-times of Aβ(1-42) aggregation (2 µM) in absence or presence of trypsin-treated EVs extracted from a.

 **Fig. S12.** **Aβ(1-42) aggregation kinetics in the presence of trypsin-control (DPBS).** Normalized aggregation kinetics data of 2 µM Aβ(1-42) in untreated or DPBS buffer subjected to the same trypsin-treatment and trypsin-removal protocol as the EV samples (see Methods for details), but in the absence of EVs (“trypsin-treated buffer”). The indicated values (9.0e8-7.2e9) correspond to the EV concentrations (particles/mL), and thus volumes, used in Fig. 4.

**Figure S13.** **Reaction parameters derived from** **Aβ(1-42) aggregation kinetics.** Lag-times (**a**) and growth-times (**b**) of Aβ(1-42) aggregation in absence or presence of untreated or heparinase-treated SHSY5Y EVs. Lag-times (**c**) and growth-times (**d**) of Aβ(1-42) aggregation in absence or presence of CHO K1 or CHO pgsA-745 EVs. The reaction parameters are derived from aggregation curves in Fig. 5.

**Table S9.** **Overall two-way ANOVA analysis of EV type and concentration on t_half_ of** **Aβ(1-42) aggregation kinetics.**

|  | Degrees of Freedom | Sum of Squares | Mean Square | F value | *p*-value |
| --- | --- | --- | --- | --- | --- |
| EV type | 1 | 8.09023 | 8.09023 | 5.89797 | 0.02732 |
| Concentration | 3 | 173.56142 | 57.85381 | 42.17676 | <0.0001 |
| Interaction | 3 | 5.25655 | 1.75218 | 1.27738 | 0.31584 |
| Model | 7 | 186.90819 | 26.70117 | 19.46577 | <0.0001 |
| Residual (Error) | 16 | 21.94718 | 1.3717 |  |  |
| Corrected Total | 23 | 208.85537 |  |  |  |

**Table S10.** **Overall two-way ANOVA analysis of EV type and concentration on t_lag_ of** **Aβ(1-42) aggregation kinetics.**

|  | Degrees of Freedom | Sum of Squares | Mean Square | F value | *p*-value |
| --- | --- | --- | --- | --- | --- |
| EV type | 1 | 0.48641 | 0.48641 | 6.4566 | 0.0218 |
| Concentration | 3 | 27.30324 | 9.10108 | 120.80833 | <0.0001 |
| Interaction | 3 | 0.60245 | 0.20082 | 2.66564 | 0.8297 |
| Model | 7 | 28.39209 | 4.05601 | 53.83979 | <0.0001 |
| Residual (Error) | 16 | 1.20536 | 0.07533 |  |  |
| Corrected Total | 23 | 29.59745 |  |  |  |

**Table S11.** **Overall two-way ANOVA analysis of EV type and concentration on t_growth_ of** **Aβ(1-42) aggregation kinetics.**

|  | Degrees of Freedom | Sum of Squares | Mean Square | F value | *p*-value |
| --- | --- | --- | --- | --- | --- |
| EV type | 1 | 15.87079 | 15.87079 | 5.95496 | 0.02669 |
| Concentration | 3 | 223.5314 | 74.51047 | 27.95745 | <0.0001 |
| Interaction | 3 | 8.46873 | 2.82291 | 1.0592 | 0.39388 |
| Model | 7 | 247.87091 | 35.41013 | 13.28642 | <0.0001 |
| Residual (Error) | 16 | 42.64221 | 2.66514 |  |  |
| Corrected Total | 23 | 290.51312 |  |  |  |

**References**

Halipi, V., N. Sasanian, J. Feng, J. Hu, Q. Lubart, D. Bernson, D. van Leeuwen, D. Ahmadpour, E. Sparr, and E. K. Esbjörner. 2024. 'Extracellular Vesicles Slow Down Aβ(1-42) Aggregation by Interfering with the Amyloid Fibril Elongation Step', *Acs Chemical Neuroscience*, 15: 944-54.
